# Wastewater-Based Genomic Surveillance of SARS-CoV-2 Antiviral Resistance Determinants in Ontario: Towards a Scalable Framework for Population-Level Antiviral Resistance Monitoring

**DOI:** 10.3390/v18080923

**Published:** 2026-08-21

**Authors:** Opeyemi U. Lawal, Valeria R. Parreira, Alyssa K. Overton, Jennifer J. Knapp, Richard Gibson, Eric J. Arts, Linkang Zhang, Fozia Rizvi, Melinda Precious, Trevor C. Charles, Lawrence Goodridge

**Affiliations:** 1 Great Lakes Institute for Environmental Research, University of Windsor, Windsor, ON N9P 3P4, Canada; 2 School of the Environment, University of Windsor, Windsor, ON N9P 3P4, Canada; 3 Canadian Research Institute for Food Safety, Department of Food Science, University of Guelph, Guelph, ON N1G 2W1, Canada; 4 Department of Biology, University of Waterloo, Waterloo, ON N2L 3G1, Canada; 5 Department of Microbiology and Immunology, Western University, London, ON N6A 3K7, Canada; 6 Metagenom Bio Life Science Inc., Waterloo, ON N2L 5V4, Canada

**Keywords:** antiviral resistance, remdesivir, sotrovimab, nirmatrelvir, wastewater surveillance, public health genomics, SARS-CoV-2, immune escape

## Abstract

Background: Wastewater surveillance has emerged as an effective tool for population-level pathogen monitoring. Its application to mutations associated with resistance to antivirals remains comparatively underdeveloped. We assessed the wastewater epidemiology framework using SARS-CoV-2 as a model pathogen to evaluate spatial, temporal, and therapeutic class-specific resistance dynamics. Methods: We analyzed about 10,000 SARS-CoV-2-positive wastewater samples from six Ontario public health regions collected between October 2021 and July 2024. Fifty-five mutations were screened, comprising therapeutic resistance-associated mutations and a biologically distinct group of immune-evasion mutations. Mutations detected in ≥10 samples at ≥1% frequency were retained for spatiotemporal analysis using LOESS smoothing and Kruskal–Wallis testing. Results: Twelve mutations met the inclusion thresholds. S:E340D, associated with reduced susceptibility to sotrovimab was geographically widespread but transient and low-frequency. Five remdesivir-associated polymerase mutations were sporadic with sharp localized peaks, including two mutations exceeding 99% frequency in isolated catchments. Three nirmatrelvir-associated protease mutations were detected, with ORF1a:Q3452K showing significant regional variation. FLiRT and FLuQE immune-evasion mutations were most persistent and abundant. LOESS smoothing showed distinct temporal patterns among mutations, while Kruskal–Wallis testing identified significant regional variation for ORF1a:Q3452K and the three immune-evasion mutations. Conclusions: These findings demonstrate that wastewater surveillance enables population-scale monitoring of antiviral resistance and immune escape-associated mutations and offers a scalable model for broader surveillance.

## 1. Introduction

Wastewater surveillance (WWS) has proven to be an effective tool for community-level tracking of infectious diseases. Throughout the COVID-19 pandemic, WWS was deployed globally to monitor SARS-CoV-2 viral load, detect emerging variants, and identify spike mutations associated with immune escape [1,2,3,4,5,6]. While WWS has been demonstrated for monitoring antibiotic resistance [7,8], its application to the surveillance of antiviral resistance remains comparatively underdeveloped. This gap is particularly important as antiviral resistance can compromise the long-term effectiveness of therapeutic strategies and undermine pandemic preparedness [9]. Clinical surveillance remains the primary approach for detecting resistance mutations, but it is constrained by limited sampling coverage, testing access, and lag times in sequencing and data sharing [10]. These limitations create the need for scalable, population-level approaches that complement clinical surveillance. Given the extensive wastewater genomic data generated during the COVID-19 pandemic and the deployment of multiple therapeutics with characterized resistance-associated mutations, SARS-CoV-2 provides a suitable model for evaluating this application of WWS.

Several antiviral therapies targeting distinct SARS-CoV-2 proteins were rapidly developed and deployed during the pandemic. Remdesivir, a viral RNA-dependent RNA polymerase (RdRp) inhibitor, was the first approved antiviral for COVID-19 and received emergency use authorization in May 2020. It was followed by molnupiravir, authorized in late 2021, and nirmatrelvir–ritonavir (Paxlovid), a main protease (Mpro) inhibitor authorized around the same period [11]. In parallel, several monoclonal antibody (mAb) therapies such as bamlanivimab, casirivimab–imdevimab, and sotrovimab were introduced to reduce severe outcomes in high-risk populations [12,13].

Shortly after clinical deployment, resistance-associated mutations were reported in both treated individuals and global genomic datasets. Mutations in the RdRp gene (nsp12), including E802D, C799Y, and M794I, have been associated with decreased susceptibility to remdesivir [14,15,16]. In the Mpro gene (nsp5), mutations such as T169I and A173T emerged under nirmatrelvir pressure and have been linked to resistance phenotypes [14,17]. Similarly, spike mutations such as S:E340D/K/A/V and S:P337L/T reduce the neutralization activity of sotrovimab [12,18,19]. More recently, S:R346T, S:F456L (FLiRT), and S:Q493E (FLuQE) have been linked to broad immune escape, limiting vaccine-elicited neutralization across multiple Omicron sublineages [20,21,22,23].

While these resistance-associated mutations have been characterized clinically, their emergence and dynamics at the population level remain poorly understood, particularly in the absence of widespread and continuous clinical sequencing. WWS has the potential to close this gap by detecting early-phase resistance emergence, regional amplification, and population-scale circulation trends. Here, we used SARS-CoV-2 as a model system to evaluate the utility of wastewater-based genomic surveillance for detecting and characterizing mutations associated with resistance to antivirals. We analyzed approximately 10,000 wastewater samples collected from six public health regions in Ontario, Canada, spanning 34 months from October 2021 to July 2024. Our goal was to characterize their temporal and regional patterns and assess WWS as a complementary population-level screening approach.

## 2. Materials and Methods

### 2.1. Sample Collection and Processing

As part of the Ontario Wastewater Surveillance Initiative, led by the Ontario Ministry of the Environment, Conservation and Parks (MECP) in collaboration with public health organizations and university partners, wastewater surveillance was conducted at 107 sampling sites across all 34 Ontario public health units, with sampling performed one to seven times weekly [4,6,24]. Participating laboratories followed established program protocols for wastewater collection and processing, SARS-CoV-2 RNA extraction and quantification, and quality assurance, including MECP-led interlaboratory proficiency testing [4,6,24]. Twenty-four-hour composite samples were collected using autosamplers or passive torpedo samplers. A subset comprising about 10,000 SARS-CoV-2-positive samples (Ct ≤ 36) collected between October 2021 and July 2024 was sequenced on Illumina platforms by participating university laboratories using a tiled-amplicon sequencing approach. Samples were grouped into six Ontario public health regions according to the public health unit associated with each sampling location [4,6,24].

### 2.2. Data Processing

Demultiplexed paired-end sequence reads were quality-filtered and trimmed to remove adapter sequences using Cutadapt v3.7 [25]. The remaining high-quality reads were mapped to the SARS-CoV-2 reference genome NC_045512.2 using minimap2 v2.28 [26], generating a BAM alignment file for each sample. Samples with incomplete metadata were excluded from downstream analysis.

### 2.3. Estimating Mutation Frequency

Mapped genomes were screened with Alcov [27] using the default minimum read depth of 10 against a manually curated 55 experimentally validated mutations that encode resistance to antiviral therapies for SARS-CoV-2 infection described in the literature, including those reported in Stanford Coronavirus Resistance Database (CoV-RDB; https://covdb.stanford.edu, assessed on 12 February 2025) [13,14,17,19,28,29,30]. Mutations associated with reduced susceptibility to remdesivir, nirmatrelvir, and sotrovimab were analyzed separately from FLiRT and FLuQE spike mutations associated with immune escape. Only mutations detected in at least 10 samples at a frequency of at least 1% were retained for spatiotemporal analysis. These thresholds were used to focus the analysis on recurrent signals and reduce interpretation of low-frequency calls, hence they were not treated as clinical resistance thresholds.

### 2.4. Statistical Analysis

All statistical analyses were conducted in R using RStudio (v1.4.1717). The individual wastewater sample served as the analytical unit, and regional comparisons were conducted without population or sampling-frequency weighting. For each retained mutation, descriptive statistics included the number of samples in which it was detected, median frequency, interquartile range, region and date of first detection, and the region, date, and frequency of peak detection. Regional heterogeneity in mutation frequency was assessed using Kruskal–Wallis tests. Pairwise regional comparisons were performed for all retained mutations using Dunn’s tests with Holm correction applied within each mutation. Temporal trends were visualized using LOESS smoothing with a span of 0.75 and pointwise 95% confidence intervals. Kruskal–Wallis *p*-values and Holm-adjusted Dunn *p*-values below 0.05 were considered statistically significant.

## 3. Results

We assessed 10,000 SARS-CoV-2 positive wastewater samples for their potential for use in tracking mutations associated with reduced susceptibility of SARS-CoV-2 to antiviral therapies and mutations associated with immune escape. The frequency of 55 unique mutations was investigated. The panel included mutations associated with nirmatrelvir, remdesivir, and sotrovimab, together with a separately classified group of FLiRT and FLuQE immune-evasion substitutions (Supplementary Table S1).

### 3.1. Spatiotemporal Characterization of the Spike Mutation S:E340D Associated with Sotrovimab Resistance

**Figure 1:**
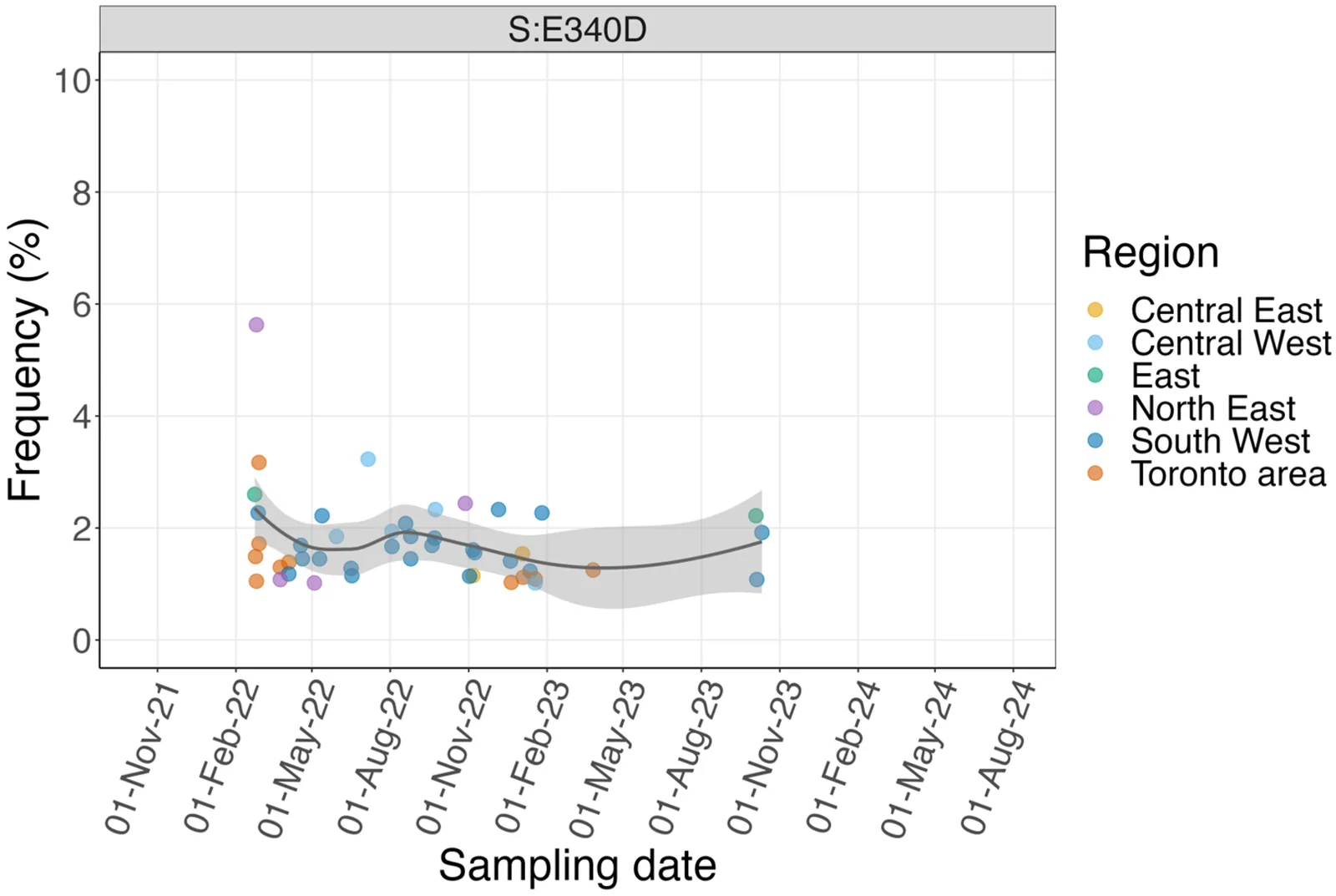
Temporal dynamics of the S:E340D mutation associated with sotrovimab resistance, detected in wastewater samples from six Ontario regions between October 2021 and July 2024. Wastewater samples were analyzed using an amplicon-based sequencing approach. Reference-based variant calling was performed against the Wuhan strain. Mutations were reported based on thresholds of a minimum read depth of 10, a relative abundance of ≥1%, and detection in at least 10 samples. The solid gray line represents the LOESS-smoothed temporal trend, and the shaded area represents the pointwise 95% confidence band.

The spike mutation S:E340D, a substitution within the receptor-binding domain (RBD) known to confer resistance to the monoclonal antibody sotrovimab, was detected in 47 wastewater samples collected between October 2021 and July 2024. This mutation was identified in all six Ontario public health regions and met established inclusion criteria (≥10 samples at ≥1% frequency). S:E340D was first detected in the East region on 23 February 2022, at a frequency of 2.6%, marking one of the earliest wastewater-documented resistance-associated spike mutations in the surveillance network (Table 1). The peak detection occurred in the North East region on 25 February 2022, reaching 5.63%, yet no single region exhibited recurrent elevation over time. Across the dataset, the mutation maintained a median frequency of 1.54% (IQR: 0.80%), with no significant regional variation (*p* = 0.161) and no instances exceeding 10% (Figure 1). The absence of persistent elevation indicates that S:E340D circulated transiently across the surveillance network. Although no catchment showed sustained dominance, its recurrence across multiple sewersheds and early detection in the surveillance timeline highlight the capacity of wastewater genomics to capture low-frequency sotrovimab resistance-associated mutations.

### 3.2. Spatiotemporal Surveillance of Remdesivir Resistance-Associated Mutations in Wastewater

**Figure 2:**
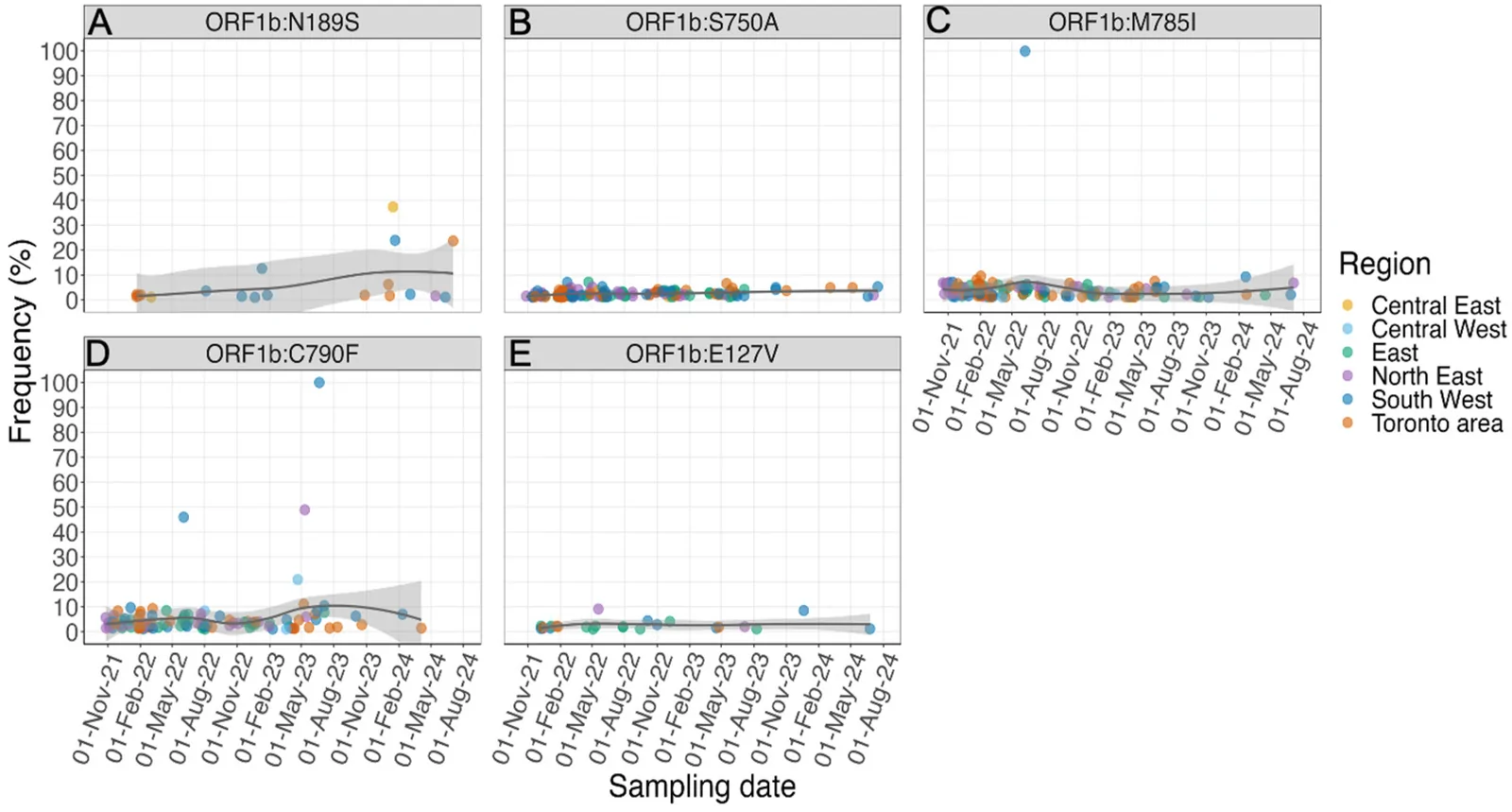
Temporal trends of mutations associated with remdesivir resistance, detected in wastewater samples from six Ontario regions between October 2021 and July 2024. Wastewater samples were analyzed using an amplicon-based sequencing approach. (**A**–**E**) Reference-based variant calling was performed against the Wuhan strain. Mutations were reported based on thresholds of a minimum read depth of 10, a relative abundance of ≥1%, and detection in at least 10 samples. The solid gray line represents the LOESS-smoothed temporal trend, and the shaded area represents the pointwise 95% confidence band.

Abundance of 12 RNA-dependent RNA polymerase (ORF1b) mutations previously implicated in reduced susceptibility to remdesivir were assessed. Of these, five mutations (ORF1b:N189S, S750A, M785I, C790F, and E127V) met predefined inclusion thresholds and were retained for quantitative assessment (Figure 2A–E). The five mutations demonstrated markedly heterogeneous detection patterns in both time and space. ORF1b:S750A was the most frequently detected (n = 142), followed by M785I (135 samples) and C790F (106 samples); N189S and E127V were detected in 21 and 24 samples, respectively. All five mutations were observed in at least four health regions, with S750A, M785I, and C790F exhibiting province-wide distribution. Median frequencies ranged from 1.89% (N189S) to 3.32% (C790F). These values, while subdominant, exceeded detection thresholds consistently and, in some instances, surged. Temporal onset varied across mutations. The earliest detections were observed in the North East region: M785I and C790F emerged in October 2021, both exceeding 5% frequency at first detection (Figure 2C,D, Table 1). In contrast, S750A first appeared shortly after (27 October 2021) at 1.51% (Figure 2B), while N189S and E127V emerged later, in Toronto (January 2022) and Eastern Ontario (December 2021), respectively (Figure 2A,E). The staggered detections describe temporal and geographic heterogeneity but do not establish independent introductions or cryptic dissemination.

Despite modest median frequencies, all five mutations exhibited isolated yet pronounced peaks in specific regions. C790F and M785I reached maximum frequencies of 100% and 99.86%, respectively, in Southwest Ontario in 2023. N189S peaked at 37.33% in Central East in early 2024, while S750A and E127V reached 7.14% (East) and 9.09% (North East), respectively. These peaks were temporally bounded, and no mutation demonstrated sustained elevation over consecutive surveillance intervals. Kruskal–Wallis tests showed no significant regional differences for any of the five mutations, with *p*-values ranging from 0.41 to 0.93. Together, these findings indicate that remdesivir resistance-associated mutations were generally detected at low-to-moderate frequencies, with transient high-frequency peaks and no consistent regional enrichment.

Notably, even mutations with province-wide detection (e.g., S750A, M785I) failed to show region-specific concentration, indicating diffusion-like patterns of circulation. Taken together, these data demonstrate that remdesivir resistance-associated mutations circulate at low-to-moderate abundance in Ontario wastewater but appear non-uniformly, transiently, and without consistent spatial anchoring. The detection of such mutations at high frequency in isolated events, approaching or reaching dominance in some catchments, could suggest localized emergence events that may carry public health significance despite their intermittent detection. These signals, inaccessible to traditional clinical sequencing frameworks, underscore the unique value of wastewater genomics for detecting resistance emergence below the clinical radar.

### 3.3. Spatiotemporal Dynamics of Main Protease Resistance-Associated Mutations Detected in Wastewater

**Figure 3:**
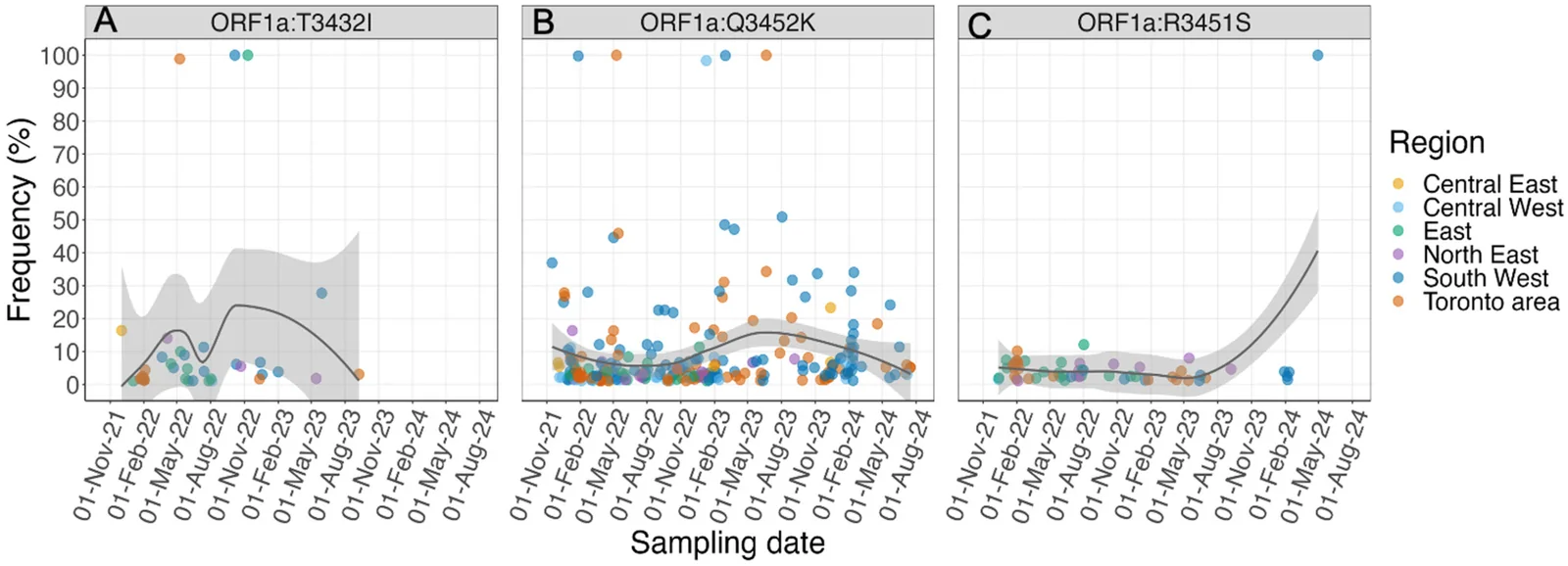
Temporal trends of mutations associated with nirmatrelvir resistance, detected in wastewater samples from six Ontario regions between October 2021 and July 2024. (**A**–**C**) Wastewater samples were analyzed using an amplicon-based sequencing approach. Reference-based variant calling was performed against the Wuhan strain. Mutations were reported based on thresholds of a minimum read depth of 10, a relative abundance of ≥1%, and detection in at least 10 samples. The solid gray line represents the LOESS-smoothed temporal trend, and the shaded area represents the pointwise 95% confidence band.

Thirty-two mutations associated with nirmatrelvir resistance were screened, of which only three ORF1a mutations, T3432I, Q3452K, and R3451S, were detected above interpretive thresholds in wastewater collected from six Ontario public health regions (Figure 3A–C). These mutations showed distinct geographic and temporal patterns, with each reaching a peak frequency of 100%. Q3452K was the most frequently observed mutation, detected in 264 samples across all six health regions. It emerged early, first appearing in South West Ontario on 16 November 2021, at a frequency of 36.90%, and reached 100% in the Toronto area on 21 June 2023 (Table 1). Its median frequency was 3.85% (IQR 6.10%), with significant regional variation (Kruskal–Wallis *p* = 0.00029). Dunn’s testing identified a significant difference between Central West and South West wastewater samples (Holm-adjusted *p* = 0.00019) (Figure 3B).

T3432I, though less frequent (38 samples), displayed a similarly punctuated trajectory. It was first detected in Central East Ontario in December 2021 with a strikingly high initial abundance (16.36%) and peaked at 100% in the East region on 10 November 2022. Despite these spikes, its overall abundance was more variable (median = 3.43%, IQR: 6.24%), and regional differences approached but did not reach statistical significance (*p* = 0.064) (Figure 3A). R3451S was detected in 60 samples across five regions and exhibited a more subdued pattern. First identified in Central West Ontario on 13 December 2021, at 1.5%, it peaked at 100% in South West Ontario in April 2024, suggesting delayed but episodic amplification. However, its lower median abundance (2.74%) and nonsignificant spatial variation (*p* = 0.263) indicate limited regional dominance or expansion (Figure 3C). Notably, all three mutations exhibited sharp, localized peaks in abundance, frequently exceeding 90%, followed by rapid dissipation. This pattern is consistent with transient selective sweeps or stochastic amplification events within sewersheds. The early detection of these mutations, often months before peak abundance, demonstrates the effectiveness of wastewater genomics for capturing resistance emergence. Their detection across disparate regions and time points also raises the possibility of convergent resistance evolution under nirmatrelvir pressure in community settings.

### 3.4. Temporal and Spatial Dynamics of FLiRT and FLuQE Immune-Evasion Mutations in Ontario Wastewater

**Figure 4:**
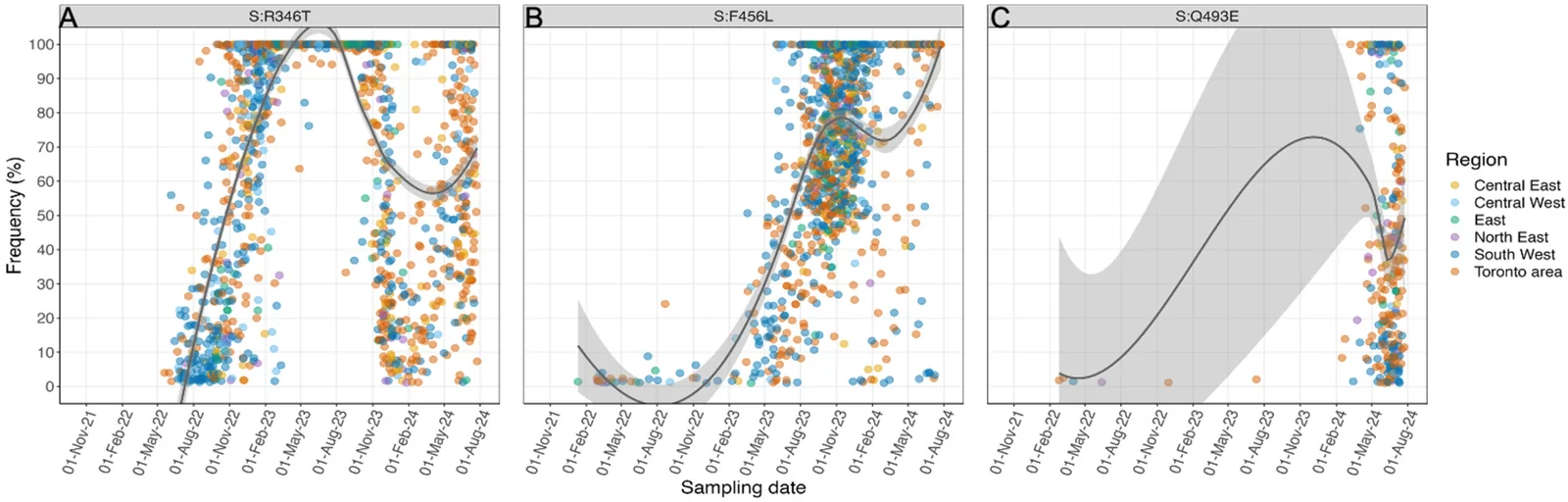
Temporal trends of mutations associated with resistance to monoclonal antibody-based immunotherapies and vaccine escape, detected in wastewater samples from six Ontario regions between October 2021 and July 2024. Wastewater samples were analyzed using an amplicon-based sequencing approach. Reference-based variant calling was performed against the Wuhan strain. FLiRT (S:R346T [**A**] and S:F456L [**B**]) and FLuQE (S:Q493E [**C**]) mutations were reported based on thresholds of a minimum read depth of 10, a relative abundance of ≥1%, and detection in at least 10 samples. The solid gray line represents the LOESS-smoothed temporal trend, and the shaded area represents the pointwise 95% confidence band.

The FLiRT (S:R346T, S:F456L) and the FLuQE (S:Q493E) mutations are associated with reduced neutralization by vaccine-elicited antibodies [22]. All three mutations were detected at high prevalence and sustained abundance in wastewater samples collected across six public health regions in Ontario between October 2021 and July 2024 (Figure 4A–C). S:R346T was the most frequently detected immune-evasion mutation and was identified in 1539 samples. It was first detected in the Toronto area on 19 May 2022, at a frequency of 4% and reached 100% in the same region on 30 September 2022 (Table 1). The mutation sustained a median frequency of 89.12% (IQR 66.61%) and varied significantly among regions (Kruskal–Wallis *p* = 0.00001). Dunn’s testing showed higher frequencies in Central East and Toronto area than East (Holm-adjusted *p* = 0.00056 and 0.0240, respectively). Frequencies were higher in South West than East, North East, and Toronto area (Holm-adjusted *p* = 0.000067, 0.0471, and 0.0351, respectively) (Figure 4A).

S:F456L was detected in 1092 samples across all six regions. It was first detected in East Ontario on 11 January 2022, at 1.39% and reached 100% in South West on 1 June 2023. Its median frequency was 74.74% (IQR 47.98%) with significant regional variation (Kruskal–Wallis *p* = 0.00001). Dunn’s testing showed higher frequencies in the Toronto area than Central East, Central West, and South West (Holm-adjusted *p* = 0.00062, 0.00323, and 0.00430, respectively) (Figure 4B). S:F456L remained detectable over an extended period and showed consistently elevated frequencies across multiple catchments (Figure 4B). Previous studies have shown that F456L reduces neutralization by vaccine-elicited sera across several Omicron sublineages, including XBB.1.5 and EG.5 [17].

S:Q493E was detected in 269 samples. It was first identified in the Toronto area on 24 February 2022, at a frequency of 1.82% and reached its highest frequency (100%) in the same region on 8 March 2024. This mutation had a median frequency of 34.75% (IQR 63.53%), with significant regional variation (Kruskal–Wallis *p* = 0.00285). Dunn’s testing showed higher frequencies in South West than Central East and East (Holm-adjusted *p* = 0.0487 and 0.0399, respectively) (Figure 4C).

Although detected less frequently than the FLiRT mutations, S:Q493E showed pronounced temporal peaks and broad geographic distribution (Figure 4C). The widespread occurrence and high frequencies of the FLiRT and FLuQE mutations distinguished their population-level patterns from those of the therapeutic resistance-associated mutations examined in this study.

## 4. Discussion

This study demonstrates the utility of wastewater-based genomic surveillance for detecting and monitoring SARS-CoV-2 mutations associated with resistance to antivirals and immune escape. The temporal and geographic patterns observed across six Ontario regions show that wastewater genomics can provide a population-level view of the occurrence and distribution of these mutations. Mutations included in the remdesivir and nirmatrelvir panels were generally detected intermittently, whereas the FLiRT and FLuQE substitutions were more persistent and abundant. This distinction is important as therapeutic resistance and immune escape arise under different biological conditions and cannot be interpreted as a single resistance phenomenon.

Among the therapeutic resistance-associated mutations tracked, those linked to remdesivir and nirmatrelvir targets exhibited divergent patterns. The RdRp mutations associated with remdesivir resistance were observed intermittently and at low-to-moderate abundance, without sustained signal dominance. Previous studies have shown that some remdesivir resistance-associated substitutions can reduce antiviral susceptibility while also affecting viral fitness, which may influence their persistence in the absence of continued drug exposure [31,32,33]. In contrast, Mpro mutations included in the nirmatrelvir panel showed pronounced temporal peaks, while ORF1a:Q3452K exhibited significant regional heterogeneity, T3432I did not reach statistical significance. The contrasting patterns indicate that mutations associated with different antiviral targets may differ in their persistence and distribution, although the biological basis of these differences was not assessed in this study.

The timing of mutation detection relative to therapeutic authorization differed among the antiviral classes. Remdesivir was authorized in Canada in July 2020 [34], and ORF1b:M785I was first detected in wastewater in October 2021. In contrast, ORF1a:T3432I was detected in December 2021 before nirmatrelvir was authorized in Canada on 17 January 2022 [35]. Sotrovimab was authorized in Canada on 30 July 2021 [36], and S:E340D was first detected in wastewater in February 2022. These differing timelines show that wastewater detection did not consistently follow therapeutic authorization and therefore cannot independently establish treatment-driven selection. Resistance-associated substitutions may already be present in circulating viral lineages or emerge during prolonged infections. Their detection therefore cannot be attributed to community antiviral use without corresponding treatment, clinical and lineage data [17,20,22,29].

The FLiRT and FLuQE spike mutations associated with immune escape showed the most consistent and sustained wastewater signals. These mutations have been associated with reduced antibody neutralization in several Omicron backgrounds [18,23,37]. S:R346T and S:F456L were detected across all six regions at high frequency and over extended periods, including frequencies reaching 100% in some samples. S:Q493E was less frequently detected but demonstrated pronounced temporal peaks and broad geographic distribution. These detection patterns occurred during phases of widespread vaccination [38,39]. The greater abundance and persistence of these immune-evasion substitutions are consistent with their occurrence in widely circulating Omicron lineages. However, the present analysis did not estimate lineage abundance or population immunity and therefore cannot determine whether their wastewater patterns resulted from lineage turnover, immune selection, or both [40,41]. Their marked difference from the less persistent therapeutic resistance-associated mutations nevertheless supports interpreting the two categories separately.

Wastewater sequencing does not resolve individual infection histories, treatment status, clinical outcomes, or patient-level resistance phenotypes, but it can reveal temporal and geographic patterns in mutation occurrence [6,42]. Since wastewater integrates signals from infected individuals regardless of symptoms, healthcare access, or testing behavior, it can identify changes across communities that may warrant targeted clinical or laboratory investigation. The detection of these mutations across multiple regions demonstrates the population-level coverage provided by the wastewater surveillance network, particularly when clinical testing and sequencing are limited. The findings support its use as a complementary screening approach rather than as a substitute for clinical resistance testing.

The findings should be interpreted within the analytical limits of wastewater amplicon sequencing. Amplicon sequencing estimates relative mutation frequencies but does not provide absolute mutant copy number. Rare mutations near the reporting threshold may be affected by sequencing error, locus-specific read depth, primer dropout, genome fragmentation, inhibitors in the wastewater matrix, and differences in sampling or laboratory workflows. Orthogonal validation by digital PCR or targeted amplicon assays was not performed and should be considered in future work for high-priority mutations. Observations showing very high mutation frequency should not be interpreted as fixation or catchment-wide lineage replacement without additional evidence. Such observations may reflect localized sampling effects, technical variation, or incomplete resolution of mixed viral populations. The absence of matched clinical genomes and regional antiviral-use data further limits the interpretation of the sources and clinical significance of the detected mutations. The analysis also focused on a predefined mutation panel rather than the abundance of circulating lineages. Integrating mutation screening with lineage deconvolution would help determine whether changes in mutation frequency coincide with the expansion or decline of lineages already carrying those substitutions.

The tracked mutations have documented associations with reduced neutralization or antiviral sensitivity but their contextual relevance may vary depending on co-circulating variants, immunity levels, and therapeutic use patterns not captured in this study [14,31]. Future integration of wastewater genomics with clinical sequencing, prescription data, and serological studies will be necessary to clarify the origins and clinical significance of the detected signals. Overall, this study demonstrates that predefined SARS-CoV-2 mutations associated with resistance to antivirals and immune escape can be monitored longitudinally across a large wastewater surveillance network. Using SARS-CoV-2 as a case study, these findings provide an empirical basis for the further development of wastewater genomics as a complementary component of population-level monitoring of mutations associated with resistance to antivirals.

## 5. Conclusions

This study demonstrates that wastewater-based genomic surveillance can be used to monitor predefined SARS-CoV-2 mutations associated with resistance to antivirals and immune escape. Using SARS-CoV-2 as a model, we tracked the temporal and geographic patterns of mutations previously linked to reduced susceptibility to antivirals and neutralizing antibodies. Mutations included in the remdesivir and nirmatrelvir panels showed different temporal and regional patterns from the more persistent FLiRT and FLuQE immune-evasion substitutions, demonstrating the importance of interpreting these mutation categories separately. This finding supports the application of wastewater genomics for population-level monitoring of antiviral resistance-associated and immune-evasion mutations as a complement to clinical genomic surveillance. Using SARS-CoV-2 as a case study, the findings provide a basis for evaluating wastewater genomics as a complementary component of antiviral resistance surveillance for other clinically relevant viral pathogens.

## Figures and Tables

**Table 1:** Summary of SARS-CoV-2 mutations’ associated resistance to antivirals and immune evasion detected in Ontario wastewater.

Mutation	Mutation Class	No. Samples	Median Abundance	* IQR	No. Region	First Detection	First Detection	First Detection Abundance	First Peak Region	First Peak Date	Peak Abundance	Kruskal–Wallis *p*
S:E340D	Sotrovimab	47	1.54	0.80	6	East	23 February 2022	2.60	North East	25 February 2022	5.63	0.16142
ORF1b:N189S	Remdesivir	21	1.89	2.15	4	Toronto area	21 January 2022	1.32	Central East	15 January 2024	37.33	0.93098
ORF1b:S750A	Remdesivir	142	2.11	1.67	6	North East	27 October 2021	1.51	East	21 April 2022	7.14	0.67205
ORF1b:M785I	Remdesivir	135	3.08	3.13	6	North East	18 October 2021	6.82	South West	7 June 2022	99.86	0.41058
ORF1b:C790F	Remdesivir	106	3.32	3.76	6	North East	26 October 2021	5.66	South West	21 June 2023	100.00	0.56394
ORF1b:E127V	Remdesivir	24	1.94	0.79	4	East	8 December 2021	2.22	North East	19 May 2022	9.09	0.92325
ORF1a:T3432I	Nirmatrelvir	38	3.43	6.24	6	Central East	2 December 2021	16.36	East	10 November 2022	100.00	0.06437
ORF1a:Q3452K	Nirmatrelvir	264	3.85	6.10	6	South West	16 November 2021	36.90	Toronto area	21 June 2023	100.00	0.00029
ORF1a:R3451S	Nirmatrelvir	60	2.74	3.94	5	Central West	13 December 2021	1.50	South West	29 April 2024	100.00	0.26266
S:R346T	Immune escape (FLiRT)	1539	89.12	66.61	6	Toronto area	19 May 2022	4.00	Toronto area	30 September 2022	100.00	0.00001
S:F456L	Immune escape (FLiRT)	1092	74.74	47.98	6	East	11 January 2022	1.39	South West	1 June 2023	100.00	0.00001
S:Q493E	Immune escape (FLuQE)	269	34.75	63.53	6	Toronto area	24 February 2022	1.82	Toronto area	8 March 2024	100.00	0.00285

* IQR = interquartile range.

## Data Availability

The raw sequence data and associated metadata supporting this study are hosted on the Canadian iMicroSeq Data Portal (https://imicroseq-dataportal.ca/ accessed on 1 August 2026) under the Ontario Wastewater Surveillance Initiative (OWSI) project and are available upon request.

## Supplementary Materials

The following supporting information can be downloaded at: https://www.mdpi.com/article/10.3390/v18080923/s1, Supplementary Table S1: Mutations associated with resistance of SARS-CoV-2 to antiviral therapies screened in the study.
