# Supplementary material for: Wastewater-Based Genomic Surveillance of SARS-CoV-2 Antiviral Resistance Determinants in Ontario: Towards a Scalable Framework for Population-Level Antiviral Resistance Monitoring
Source: Viruses. 2026 Aug 21;18(8):923. doi: 10.3390/v18080923 (PMC13517777; doi:10.3390/v18080923)
Supplement: Supplementary file 1 [file viruses-18-00923-s001.zip › viruses-4418296-supplementary.pdf]

Supplementary Table S1 – Mutations associated with resistance of SARS-CoV-2 to antiviral therapies screened in the study ¶.

Nirmatrelvir resistance	Remdesivir resistance	Sotrovimab resistance	FLiRT mutation	FLuQE mutation
ORF1a:A3436T	ORF1b:C455Y	S:E340D	S:R346T	S:Q493E
ORF1a:A3436V	ORF1b:V783I		S:F456L	
ORF1a:A3457S	ORF1a:N2187S			
ORF1a:E3429A	ORF1a:T3058I			
ORF1a:E3429G	ORF1b:V157A			
ORF1a:E3429K	ORF1b:N189S			
ORF1a:E3429Q	ORF1b:A367V			
ORF1a:E3429V	ORF1b:D475Y			
ORF1a:F3403L	ORF1b:S750A			
ORF1a:F3403S	ORF1b:M785I			
ORF1a:G3406S	ORF1b:E787G			
ORF1a:H3435L	ORF1b:C790R			
ORF1a:H3435N	ORF1b:C790F			
ORF1a:H3435Q	ORF1b:C790Y			
ORF1a:H3435Y	ORF1b:E793A			
ORF1a:L3430F	ORF1b:E793D			
ORF1a:M3428R	ORF1b:E127V			
ORF1a:M3428T				
ORF1a:P3515L				
ORF1a:Q3452K				
ORF1a:Q3455E				
ORF1a:Q3455H				
ORF1a:Q3455K				
ORF1a:Q3455L				
ORF1a:Q3455P				
ORF1a:Q3455R				
ORF1a:R3451G				
ORF1a:R3451S				
ORF1a:S3407A				
ORF1a:S3407L				
ORF1a:S3407P				
ORF1a:T3567I				
ORF1a:T3432I				
ORF1a:V3560A				

¶ Experimentally validated mutations that encode resistance to antiviral therapies for SARS-CoV-2 infection described in literature [13,14,17,19,29–31] and including those reported in Stanford Coronavirus Resistance Database (CoV-RDB; https://covdb.stanford.edu).
